# Decreasing Prevalence of Transfusion Transmitted Infection in Indian Scenario

**DOI:** 10.1155/2014/173939

**Published:** 2014-01-27

**Authors:** Tulika Chandra, S. Nishat Fatima Rizvi, Devisha Agarwal

**Affiliations:** Department of Transfusion Medicine and Blood Bank, King George's Medical University, Lucknow, Uttar Pradesh 226003, India

## Abstract

Transfusion transmitted infections are major problem associated with blood transfusion. Accurate estimates of risk of TTIs are essential for monitoring the safety of blood supply and evaluating the efficacy of currently employed screening procedures. The present study was carried out to assess the percentage of voluntary donors and replacement donors and to find out prevalence and changing trends of various TTIs blood donors in recent years. A study was carried out on blood units of voluntary and replacement donors which were collected from January 2008 to December 2012. On screening of 180,371 replacement units, seropositivity of transfusion transmitted disease in replacement donors was 0.15% in HIV, 1.67% in hepatitis B surface antigen, 0.49% in hepatitis C virus, 0.01% in VDRL, and 0.009% in malaria. Of 11,977 voluntary units, seropositivity of transfusion transmitted disease in voluntary donors was 0.08% in HIV, 0.24% in hepatitis B surface antigen, 0.001% in hepatitis C virus, 0.008% in VDRL (sexually transmitted disease), and 0.01% in malaria. From results it has been concluded that prevalence of transfusion transmitted infection (HIV, HBV, HCV, VDRL, and malaria) was more in replacement donors in comparison to voluntary donors. Extensive donor selection and screening procedures will help in improving the blood safety.

## 1. Introduction

Transfusion transmitted infections (TTIs) are a major problem associated with blood transfusion. Accurate estimates of the risk of TTIs are essential for monitoring the safety of blood supply and evaluating the efficacy of the currently employed screening procedures “as discussed by Gupta et al. [1].”

Transfusion transmitted infections (TTI) are a great concern of safety for patients. The magnitude of the TTI varies from country to country depending on TTIs' loads in that particular population from where blood units are sourced. Multiple measures are taken to minimize TTI transmission in the respective population. The majority of the problems are due to the prevalence of asymptomatic carriers in the society, as well as blood donations during the window period of infections. Concealing of medical history by captive, paid, or professional blood donors, who widely exist in developing countries, also poses a great threat to safe blood supply. There is a long list of viruses, parasites, and bacteria, which can be transmitted through blood transfusions. Among them, important transfusion transmitted viruses are human immunodeficiency virus (HIV-I/II), hepatitis B virus (HBV), hepatitis C virus (HCV), syphilis infection by Spirochaetes, and transfusion associated malaria infection “as discussed by Choudhury [2].”

For a safe blood service in our country, where comprehensive laboratory tests are neither possible nor pragmatic, it is best to switch over to 100% voluntary donations. The key to recruiting and retaining safe blood donors is good epidemiological data on the prevalence (and incidence, where possible) of infectious markers in the general population to identify low-risk donor populations coupled with an effective donor education, motivation, and recruitment strategy to recruit new voluntary not remunerated blood donors from these populations “as discussed by NCMH Background papers [3].”

The present study was carried out to assess the percentage of voluntary donors and replacement donors and to find out the prevalence and changing trends of various TTIs blood donors in recent years.

## 2. Material and Methods

The study was carried out at the Department of Transfusion Medicine, King George's Medical University, Lucknow. Donors were carefully screened by trained personnel after a complete physical examination and satisfactorily answering the donor's questionnaire. Replacement donors were those who donated blood in exchange for receiving blood units for their patients. Voluntary blood donors donated blood without incentive for the cause. Written consent was also taken from them prior to donation. A total of 192,348 blood units (Voluntary and replacement) were collected from January 2008 to December 2012. From these units, 5 mL donor samples were obtained for serological testing. Samples were collected in test tubes at the time of bleeding, and blood was screened for hepatitis B surface antigen (HBsAG) by SD HBsAg Kit, hepatitis C virus (HCV) by SD HCV Kit, and human immunodeficiency virus (HIV) by SD HIV kit, Bio Standard Diagnostic in the Transfusion Transmitted Diseases Screening Laboratory at the Department of Transfusion Medicine, KGMU, Lucknow. These were third generation enzyme-linked immunoadsorbent assay technique (followed by standard protocol). Syphilis was tested by RPR-Rapid Plasma Reagin Kit (modified slide test, SPAN Diagnostic) and Malaria was screened by SD Malaria Kit (one step, rapid immunochromatographic test for the simultaneous detection of malaria, Bio Standard Diagnostic).

## 3. Results

A total of 192,348 blood units were received in our blood bank, out of which 180,371 were replacement donations and 11,977 were Voluntary donations. On screening of 1,80,371 replacement units, 279 donors (0.15%) were seropositive for the HIV infection ranging from 0.03% to 0.27% (range of percentage), 3029 donors (1.67%) were seropositive for HBV infections ranging from 1.33% to 2.94% (range of percentage), 885 donors (0.49%) were seropositive for HCV infections ranging from 0.09% to 1.71% (range of percentage), 27 donors (0.01%) were seropositive for VDRL (veneral disease) ranging from 0.01% to 0.04% (range of percentage) and 17 donors (0.009%) were seropositive for Malaria ranging from 0.005% to 0.02% (range of percentage) (Table 1). Of 11,977 voluntary blood donors, 10 (0.08%) were seropositive for HIV infections ranging from 0.03% to 0.17% (range of percentage), 29 (0.24%) were seropositive for HBV infections ranging from 0.16% to 0.34% (range of percentage), 15 (0.001%) were seropositive for HCV infections ranging from 0.001% to 0.25% (range of percentage), 1 (0.008%) were seropositive for VDRL infection and 2 (0.01%) was seropositive for malaria infection (Table 2).

## 4. Discussions

Disease burden estimations based on sound epidemiological research provide the foundation for public policy. Accurate estimates of risk of TTIs are essential for monitoring the safety of blood supply and evaluating the efficacy of the currently employed screening procedures “as discussed by NACO [4].”

The prevalence of TTIs among blood donors in well-structured health care system with a well-organized blood establishment can be used as a reliable tool for statistical estimations of those infectious agents that can be transmitted through blood products and can contribute to statistical estimation of these viruses in the general population “as discussed by Gharehbaghian [5].”

A study shows that a total of 1600 donors were tested, of these 113 (7.06%) were reactive for transfusion transmitted infections. This comprised 50 (3.12%) cases positive for the presence of HbsAg, 23 (1.43%) cases positive for the presence of HCV Ab, 6 (0.37%) cases positive for the presence of HIV, 32 (2%) cases positive for VDRL, and 2 (0.12%) cases for gametocyte of Plasmodium falciparum “as discussed by Shahtaj et al. [6]”. In Iran, the results of serological screening tests for HBV, HCV, HIV, and syphilis infections performed by Tehran blood transfusion service between 2003 and 2005 in 1004889 subjects showed that the seroprevalence was 0.9% for HBsAg, 2.1% for anti-HCV, 0.2% for HIV Antibody 1 and 2, and 0.04% for VDRL. Between 2003 and 2005, a decreasing trend was observed in the frequency of HbsAg “as discussed by Khedmat et al. [7].” Jeremiah et al. reported HCV prevalence rates of 5.0% among donors in Port Harcourt, Nigeria [8].

Very low prevalence rates of replacement and voluntary donors for HIV (0.08-0.15%), HBV (0.24–1.67%), HCV (0.001–0.49%), VDRL (0.008–0.01%), and malaria (0.009–0.01%) in our study had been found.

The difference in infection rates between voluntary and replacement donors has been observed in many earlier studies “as discussed elsewhere [9–11].” A study shows that a rate of transfusion transmitted malaria varies from less than 0.2 in nonendemic countries to 50 or more cases per million in endemic countries “as discussed by Mollison et al. [11].” A study conducted at Agha Khan University Hospital showed that the rate of seropositivity of syphilis does not seem to be changing during the past several years, with annual rate of less than 1%. Of the 114,122 samples tested during the mentioned period, 252 donors were positive for syphilis antibodies making an overall prevalence of 0.22% “as discussed by Moiz et al. [12].” Acute and chronic viral hepatitis are common public health problems in Pakistan and associated with serious complications. Data regarding the prevalence of hepatitis B and C virus infections among healthy blood donors is well established in Karachi, Rawalpindi, Islamabad, Faisalabad, Lahore and Abbottabad. Data regarding the epidemiology of HIV infection among blood donors is not available at most of the blood transfusion centers. Blood Transfusion Center Nishtar Hospital Multan and Fatimid Blood Transfusion Center Multan tested for HbsAg, anti-HCV and HIV and noted that prevalence of hepatitis B and C and HIV infection was 3.37%, 0.27%, and 0%, respectively, “as discussed by Mahmood et al. [13].” The reported prevalence figures for HBsAg and anti-HCV in other studies are quite variable, depending upon screening protocol, study groups selected, and methodology of testing. Donor's selection processes are intended to assess potential blood donors on the basis of their risk of TTIs, temporarily or permanently deferring those who report potential exposures and so reducing the infective risks of transfusion “as discussed by Mosley [14].”

In our study the seropositivity of VDRL infection is low 0.01% which has been constant in last 12 years from 2001–2012. And the seropositivity of Malaria is also low (0.009%). Prevalence of HIV infection is 0.15% in 2008-2012 but it was higher in 2001-2007 which was (0.23%) “as discussed by Chandra et al. [9]”. The overall seroprevelance of HBV and HCV from 2008–2012 had been decreased to 1.67% and 0.49% but it had been increased significantly (2.26–2.94%) and (0.72–1.71%) in 2008-2009 while in our earlier study from (2001–2007), the overall seroprevalence of HBV and HCV infection among blood donors was 1.96% and 0.85% respectively “as discussed by Chandra et al. [9]”.

From the results it has been concluded that prevalence of transfusion transmitted infection (HIV, HBV, HCV, VDRL and malaria) was higher in replacement donors in comparison to voluntary donors. Extensive donor selection and screening procedures will help in improving the blood safety. In all the markers tested there was increased prevalence of TTI among the replacement donors as compared to voluntary donors. The prevalence of HIV has been decreasing in the Indian population supporting the growing awareness of this life threatening diseases. HBsAg infection still continues to be a menace to the society because, in spite of decreasing trend, incidence of the disease is still very high in general population. There has been a significant reduction in the prevalence of HCV, probably due to the availability of diagnostic kits as well as increased awareness among the blood donors. Malaria and VDRL appear to be sporadic findings and detection is mainly based on sensitive kits available.

## Figures and Tables

**Table 1:** Prevalence of transfusion transmitted infections among replacement blood donors (2008-2009).

No.	Year	Total no. of samples	HIV (%)	HBV (%)	HCV (%)	VDRL (%)	Malaria (%)
1	2008	23468	64 (0.27%)	690 (2.94%)	403 (1.71%)	8 (0.03%)	Nil
2	2009	28340	72 (0.25%)	642 (2.26%)	206 (0.72%)	14 (0.04%)	Nil
3	2010	37281	54 (0.14%)	478 (1.28%)	128 (0.34%)	5 (0.01%)	Nil
4	2011	38844	72 (0.18%)	519 (1.33%)	98 (0.25%)	Nil	2 (0.005%)
5	2012	52438	17 (0.03%)	700 (1.33%)	50 (0.09%)	Nil	15 (0.02%)

	Total	180371	279 (0.15%)	3029 (1.67%)	885 (0.49%)	27 (0.01%)	17 (0.009%)

HBV: hepatitis B virus; HCV: hepatitis C virus; VDRL: Venereal Disease Research Laboratory.

**Table 2:** Prevalence of transfusion transmitted infections among voluntary blood donors (2008-2009).

No.	Year	Total no. of samples	HIV (%)	HBV (%)	HCV (%)	VDRL (%)	Malaria (%)
1	2008	1176	2 (0.17%)	4 (0.34%)	3 (0.25%)	Nil	Nil
2	2009	1611	2 (0.12%)	4 (0.24%)	2 (0.12%)	Nil	Nil
3	2010	2256	3 (0.13%)	7 (0.31%)	3 (0.13%)	1 (0.04%)	Nil
4	2011	3191	1 (0.03%)	8 (0.25%)	4 (0.12%)	Nil	Nil
5	2012	3743	2 (0.05%)	6 (0.16%)	3 (0.08%)	Nil	2 (0.05%)

	Total	11977	10 (0.08%)	29 (0.24%)	15 (0.001%)	1 (0.008%)	2 (0.01%)

HBV: hepatitis B virus; HCV: hepatitis C virus; VDRL: Venereal Disease Research Laboratory.
